# Feasibility and workflow efficiency of automated deep inspiration breath-hold for locoregional breast irradiation on a ring-gantry accelerator

**DOI:** 10.1016/j.phro.2026.100904

**Published:** 2026-01-13

**Authors:** Sarra Midani, Paul Retif, Sébastien Maksimovic, Clémence Bondue, Mohammed Yacoubi, Gianandrea Pietta, Anwar Al Salah, Estelle Pfletschinger, Motchy Saleh, Abdourahamane Djibo Sidikou, Romain Letellier, Fabian Taesch, Emilie Verrecchia-Ramos, Xavier Michel

**Affiliations:** a Radiation Therapy Department, CHR Metz-Thionville, Metz, France; b Medical Physics Unit, CHR Metz-Thionville, Metz, France; c Université de Lorraine, CNRS, CRAN, F-54000 Nancy, France

## Abstract

**Purpose:**

To evaluate the feasibility, dosimetric quality, workflow efficiency, and early tolerance of automated deep-inspiration breath-hold (DIBH) breast radiotherapy delivered on a ring-gantry platform.

**Materials and methods:**

Twenty patients requiring locoregional irradiation were treated on a Radixact ring-gantry system between February and September 2025 using a static-beam intensity-modulated technique in automated DIBH. Dose/volume metrics for targets and organs of interest (OOIs), workflow parameters, and acute side effects were collected. Benchmark helical tomotherapy plans in DIBH conditions were reoptimized for comparison.

**Results:**

All patients completed DIBH treatment. PTV coverage was consistently achieved (mean V_95%_: 97.2% for low-risk and 99.2% for boost volumes) and OOI objectives were met. Daily image acquisition required 20–32 s. Median expected beam-on time was 230 s, while delivered beam-on time was 416 s. Median fraction duration was approximately 10 min, including setup, imaging and delivery. A total of 3511 gated beam segments were recorded (median duration 1.8 s), confirming reproducibility and patient compliance. Compared with helical delivery in a theoretical DIBH scenario, static-beam IMRT method reduced contralateral exposure, while helical delivery yielded slightly lower cardiac doses; planned beam-on times were significantly longer with helical mode (+54%). Acute side effects were limited to grade 1 (60%) or 2 (10%) dermatitis and grade 1 esophagitis (15%), with no grade ≥3 events at median 2 months.

**Conclusions:**

Fully automated DIBH breast radiotherapy on a ring-based accelerator is feasible, safe and compatible with routine workflow. This study provides the first experience supporting automated DIBH gated delivery on a ring-based accelerator.

## Highlights

• Study performed on twenty patients receiving locoregional breast irradiation.
• Median fraction duration was 10 min, including setup, imaging and delivery.
• Over 3500 reproducible breath-holds confirmed patient compliance and feasibility.
• Only mild to moderate early side effects observed and no severe reactions.

## Graphical abstract

**Figure:**
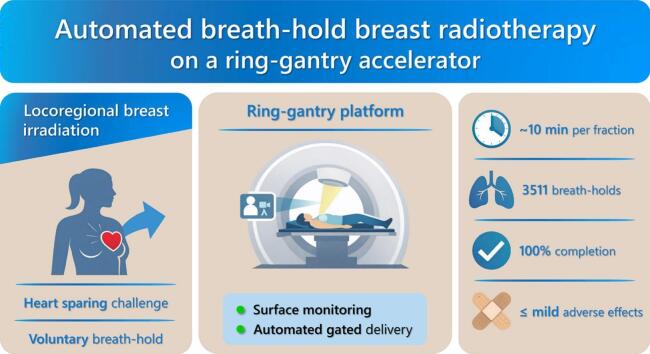

## 1 Introduction

Breast cancer is the most common malignancy in women worldwide, and radiotherapy remains a cornerstone of curative treatment after both breast-conserving surgery and mastectomy [1], [2]. While modern techniques ensure excellent local control, minimizing radiation dose to organs of interest (OOIs) – particularly the heart, lungs, and contralateral breast – remains a major concern [3], [4], [5]. Cardiotoxicity is especially relevant in left-sided breast irradiation, where long-term studies have demonstrated a linear increase in ischemic heart disease risk with rising mean heart dose [6], [7], [8], [9], [10]. In breast cancer radiotherapy, deep-inspiration breath-hold (DIBH) typically reduces cardiac exposure by increasing the heart–chest wall separation. Reported reductions in mean heart dose generally range from 20% to 40%, depending on patient anatomy and the inclusion of regional lymphatics, while also decreasing ipsilateral lung dose [11], [12], [13], [14].

Over the past two decades, DIBH techniques have evolved from manual beam-holds triggered by therapists, to spirometry- or infrared-based monitoring, and more recently to surface-guided radiotherapy (SGRT), which offers non-invasive monitoring and real-time gating [15], [16], [17], [18], [19], [20], [21], [22]. Most clinical implementations have been developed for C-arm linear accelerators (LINACs), where automation is available but often limited, requiring therapist intervention and exposing patients to collision risks due to gantry geometry [23], [24]. Ring-based LINACs provide mechanical advantages such as reduced collision risk and simplified clearance, but to date, very limited clinical data have yet been published regarding fully automated gating on ring-gantry platforms [25], [26], [27], [28].

A new generation of ring-gantry radiotherapy platforms now integrates automated deep-inspiration breath-hold workflows. These systems combine in-bore surface tracking with direct gating control, enabling automated beam on/off transitions without therapist intervention, improving workflow efficiency and reducing the risk of human error [29], [30]. A comprehensive end-to-end technical validation of this automated breath-hold workflow on a ring-gantry platform was recently reported, demonstrating sub-millimetric accuracy, high dosimetric robustness, and latencies well below AAPM TG-147 recommendations, confirming the technical readiness of the system for clinical use [31], [32].

Building on this foundation, the present study reports the first clinical evaluation of automated DIBH on a ring-based accelerator. Specifically, we investigated the feasibility of locoregional breast irradiation using static intensity-modulated radiotherapy (IMRT) beams in DIBH on a ring-gantry platform, focusing on patients requiring complex target volumes including the internal mammary and supraclavicular lymph nodes. The objectives were to assess target coverage and OOI sparing, evaluate treatment workflow metrics including imaging and delivery times, and report early clinical tolerance.

## 2 Materials and methods

### 2.1 Patient selection

Between February and September 2025, 20 consecutive patients requiring locoregional breast irradiation (whole breast or chest wall including internal mammary and supraclavicular/axillary nodes) were treated with DIBH on the Radixact (Accuray, Sunnyvale, USA) platform for a total of 320 fractions. One underwent bilateral irradiation; all others had left-sided disease. According to institutional policy, this retrospective observational study did not require approval by a local ethics committee. All patients provided informed consent. Prescriptions were 15 × 2.67 Gy, 15 × 3.2 Gy with simultaneous boost (according to NRG RTOG 1005), or 25 × 2 Gy after breast-conserving surgery or mastectomy (Table 1) [33].

**Table 1:** Descriptive patient characteristics and radiotherapy parameters.

	N	(%)
Patients who completed DIBH treatment	20	(100%)
Fractionation schedule		
15 × 2.67 Gy	14	(70%)
15 × 3.2 Gy (simulatenous integrated boost)	4	(20%)
25 × 2 Gy	2	(10%)
Breast	11	(55%)
Chest wall	9	(45%)
Laterality		
Left	19	(95%)
Right	0	(0%)
Bilateral	1	(5%)
Sex		
Female	20	(100%)
Male	0	(0%)
Age at start radiotherapy (years)		
Median	54	
Range	31–83	
Body mass index		
Median	26.3	
Range	16.4–34.8	

### 2.2 Treatment preparation

Patients were positioned supine on an AIO 2.0 breast board (Orfit, Wijnegem, Belgium) with a 5° inclination foam mounted on a 3D-printed add-on to fit within the Radixact bore. Arms were raised above the head, with a personalized chin mask for immobilization. A radio-opaque wire was placed along breast borders and surgical scar to aid delineation. Planning CT scans were acquired in DIBH (3-mm slices, 120 kV). DIBH was monitored with the Sentinel (C-RAD AB, Uppsala, Sweden) system and supported by in-room visual coaching with light panels. Patients underwent training to ensure reproducibility, with target breath-hold duration of 20–30 s. The amplitude was set at 80% of maximum inspiration, with a gating window of ±2.5 mm.

Clinical target volumes included the breast/chest wall, internal mammary chain, and supraclavicular/axillary regions and were delineated according to European Society of Radiation Oncology (ESTRO) consensus guidelines and French national recommendations for breast radiotherapy [34], [35]. OOIs (lungs, heart, left anterior descending artery (LAD), contralateral breast, and spinal cord) were contoured following the same national recommendations. Planning Target Volumes (PTVs) were generated with a 5-mm isotropic margin, minus 2 mm from the body surface.

Plans were created with the Precision (Accuray, Sunnyvale, USA) treatment planning system using TomoDirect static IMRT beams optimized with VOLO Ultra [36]. Typical configurations used 6 fixed beams, composed of two tangential fields complemented by four evenly distributed intermediate beams. Additional anterior-oblique fields were included when necessary to enhance nodal coverage. Parameters included 5.0 cm dynamic jaws, pitch 0.5, and a ∼2 cm skin flash. Planning goals required ≥95% of the PTV to receive ≥95% of the prescribed dose, while meeting institutional and international OOI dose constraints [35], [37], [38], [39] (Supplementary Table S1). A target beam-on time <200–250 s was prioritized when possible.

For benchmarking, all TomoDirect treatment plans were reoptimized in helical mode (5.0 cm jaws, pitch ≈0.28) on the DIBH planning CT according to local protocols. No virtual blocks were used for contralateral organ sparing. Instead, contralateral breast and lung sparing was achieved through optimization objectives, including constraints on mean dose and maximum dose to both organs. Plans were required to meet target coverage objectives, and OOI doses and beam-on times were compared.

### 2.3 Image guidance and treatment delivery

Daily setup was performed with free-breathing surface alignment followed by DIBH kVCT (ClearRT; 20–32 s acquisition, 1.8 mm slices) and registration to bone and soft tissue [40], [41]. VitalHold, the integrated SGRT–based automated gating functionality of the Radixact system, provided continuous in-bore surface monitoring with automated gating using the Catalyst^+^ HD (C-RAD AB, Uppsala, Sweden) surface-guidance system connected to the Radixact accelerator. Patients were instructed to maintain the breath-hold as long as possible and manage their own breathing cycles during treatment.

### 2.4 Data collection and analysis

Recorded data included patient age, body mass index, side effects (dermatitis, esophagitis), PTV and lung volumes, dosimetric indices (V_95%_, D_98%_, D_2%_, Homogeneity Index (HI) = D_2%_/D_98%_ for PTVs; Dose–volume histograms (DVH) metrics for OOIs), workflow metrics (setup, imaging, total and individual beam-on times, fraction duration). Fraction duration was extracted from the information system as the time elapsed between the moment the treatment plan was opened and then closed on the treatment console.

Given the limited cohort size and the non-normal distribution of several workflow variables, descriptive statistics such as treatment time metrics were reported as median values. Dosimetric parameters, which exhibited near-normal distributions, were summarized using mean ± standard deviation (SD). Differences between TomoDirect and reoptimized helical treatment plans were assessed using paired t-tests (Minitab 16.2.1, Minitab LLC, State College, PA, USA). A p-value <0.05 was considered significant.

## 3 Results

PTV coverage objectives were achieved for all 20 patients. In the subgroup of 17 patients treated with the 15-fraction schedule for unilateral breast irradiation, the following dosimetric results were obtained: for the low-risk PTV, the mean V_95%_ was 97.2% ± 1.3% (range, 95.4–99.4%). Among patients without boost, the mean HI was 1.10 ± 0.02 (range, 1.08–1.15). In patients receiving a boost, the high-risk PTV achieved a mean V_95%_ of 99.2% ± 1.3% (range, 97.2–100%) with a mean HI of 1.05 ± 0.02 (range, 1.03–1.07). The average low-risk PTV volume was 946 ± 490 cm^3^ (range, 264–1959 cm^3^).

**Fig. 1:**
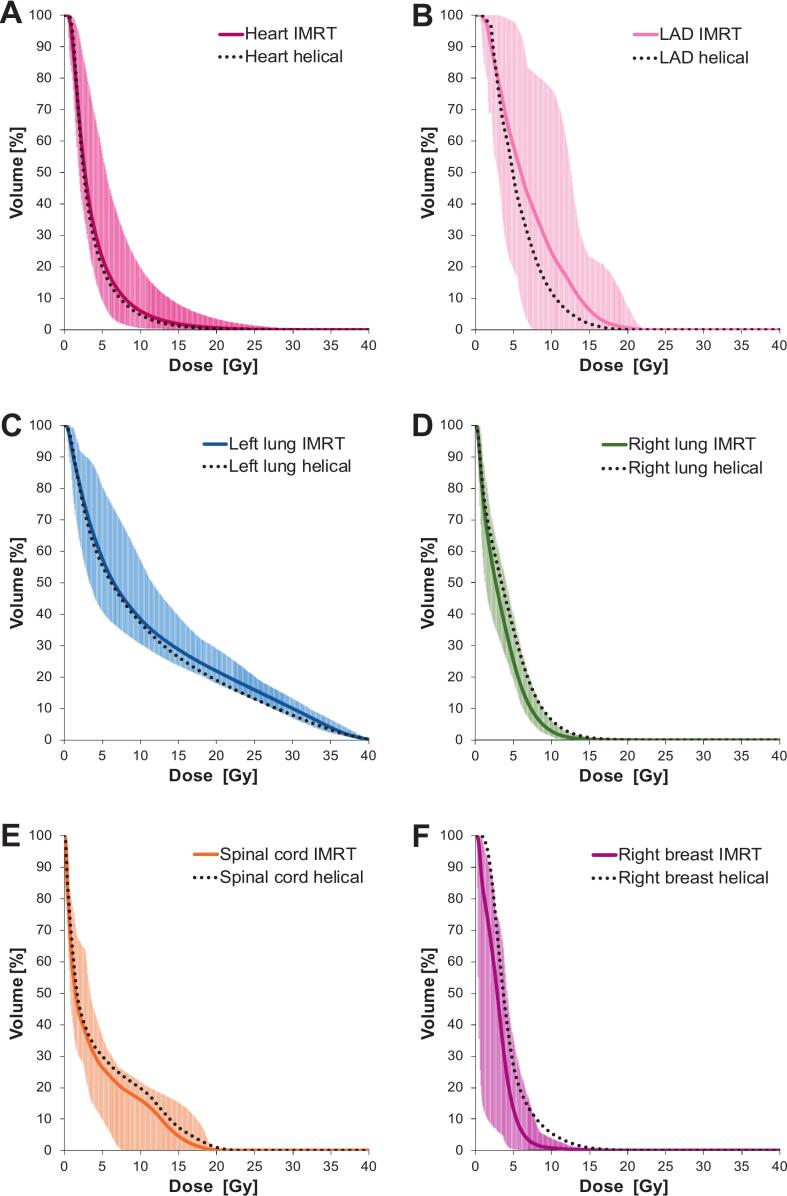
DVH of organs of interest for 17 patients treated with static-beam IMRT mode in DIBH using the 15-fraction schedule for unilateral breast irradiation. For each organ, the solid line represents the mean DVH across all patients, while the shaded area indicates the minimum and maximum values. (A) Heart, (B) left anterior descending artery (LAD), (C) ipsilateral lung (left), (D) contralateral lung (right), (E) spinal cord, (F) contralateral breast. Doted black lines represents the mean DVH for the helical delivery DIBH scenario.

All OOI constraints were respected. For the 15-fraction subgroup, mean heart dose was 3.9 ± 1.1 Gy, with V_17Gy_ of 1.3% ± 1.4%. The LAD received 7.0 ± 2.2 Gy on average (D_2%_ 14.9 Gy, maxima <20 Gy). The ipsilateral lung mean dose was 11.2 ± 1.1 Gy with V_17Gy_ 25.8%, while contralateral lung and breast doses remained low (3.4 and 3.0 Gy, respectively). The mean maximum spinal cord dose was 16.9 Gy. Fig. 1 illustrates DVHs for all OOIs.

**Fig. 2:**
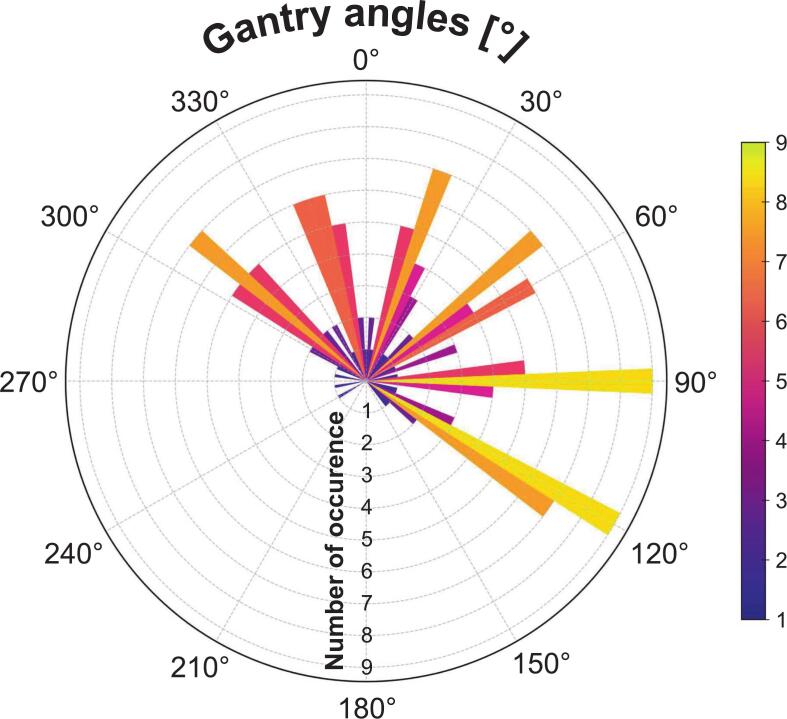
Polar distribution of gantry angles selected for IMRT beams. Bars represent the frequency of occurrence for each 5° interval across all patients, with the color scale encoding the number of occurrences. Tangential beam orientations (≈120° and 300°) predominated, complemented by recurrent anterior positions (≈345°, 15°, 50°, 90°) and occasional oblique beams for nodal coverage.

Most plans used six beams, typically tangential around 120° and 300°, with additional fields (345°, 15°, 50°, 90°) for nodal coverage. Beam angle distribution is shown in Fig. 2.

**Fig. 3:**
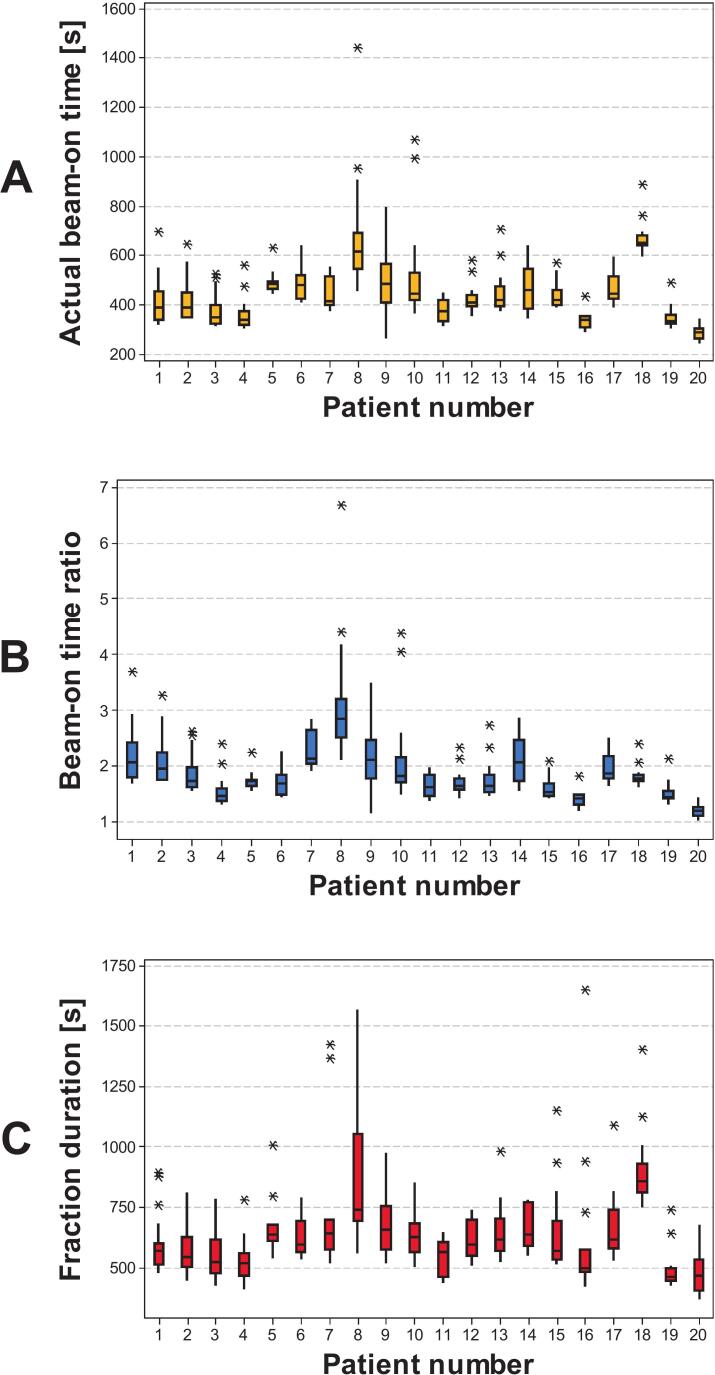
Treatment time analysis across patients. (A) Actual beam-on time [s] measured during DIBH delivery. (B) Ratio between actual and expected beam-on times. (C) Total fraction duration [s], including setup, imaging, and beam delivery. Data are shown as box plots for each patient showing median, interquartile range, and minimum/maximum values; outliers are represented as individual markers.

**Fig. 4:**
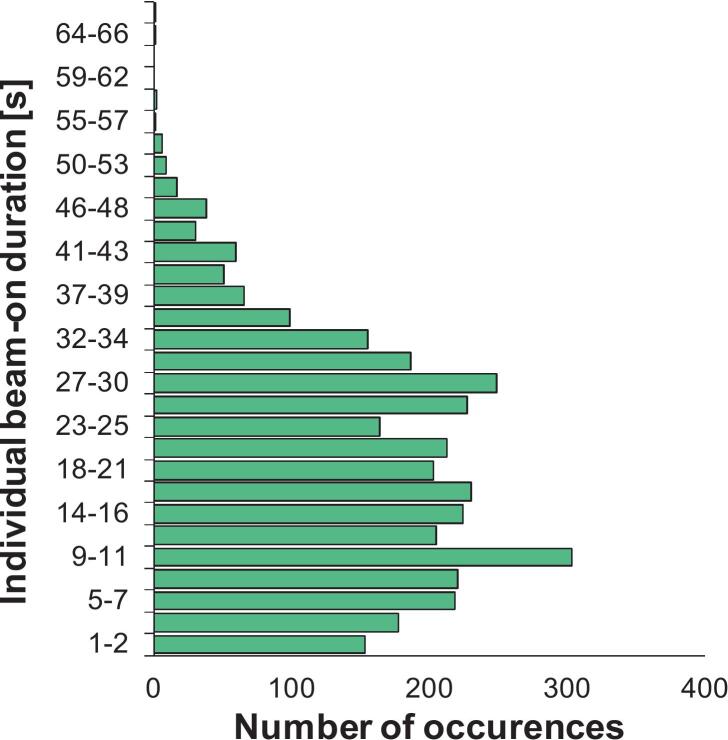
Distribution of individual beam-on occurrences, defined as breath-hold periods combined with beam delivery, recorded during treatment (N = 3511). Most occurrences lasted between 7 and 35 s, while longer durations above 50 s were less frequent.

The acquisition time of kVCT images ranged from 20 to 32 s. The median planned beam-on time was 230.5 s (range, 183.9–367.8 s). The longest value was observed in patient #18, who underwent bilateral irradiation. The median actual beam-on time, measured during DIBH delivery, was 416.0 s (range, 243.0–1443.0 s). The median ratio between actual and planned beam-on times was 1.8 (range, 1.0–6.7). The median total fraction duration was 590.5 s (range, 367.0–1651.0 s). Individual patient data are summarized as box plots in Fig. 3. Across all treatment fractions, a total of 3511 individual beam-on periods (i.e., combinations of breath-hold and irradiation) were recorded. The median duration was 18.5 s (range, 0.8–69.3 s). The majority of individual beam-ons lasted between 7 and 35 s, with fewer events exceeding 50 s. The median number of individual beam-ons per fraction was 10 (range, 5–29) (Fig. 4).

Both static-beam IMRT and helical delivery modes achieved clinically acceptable treatment plans. Helical delivery method slightly reduced mean heart (3.6 ± 1.0 vs. 3.9 ± 1.1 Gy) and LAD doses (5.7 ± 1.5 vs. 7.0 Gy ± 2.2), while static-beam IMRT mode significantly lowered contralateral lung (3.4 ± 0.4 vs. 4.2 ± 0.8 Gy) and breast doses (3.0 ± 0.8 vs. 4.4 ± 0.9 Gy). For the low-risk target volume, the HI favored helical delivery method, while both techniques performed similarly for the high-risk PTV. Expected beam-on times were higher for helical mode (median values: 355 vs. 230 s, +54%). Results are summarized in Table 2, Fig. 1 shows mean DVH for each OOI and Fig. S1 illustrates a typical dose distribution comparison between both techniques.

**Table 2:** Comparison of mean dosimetric parameters (±SD) for target volumes and organs at risk between static-beam IMRT and helical delivery modes. Statistically significant differences are marked with ** (p < 0.05).

Organ at risk	Metric	TomoDirect (mean ± SD)	Helical (mean ± SD)	p-value
High-risk PTV	HI	1.05 ± 0.02	1.06 ± 0.01	0.418
Low-risk PTV	HI	1.10 ± 0.02	1.07 ± 0.01	0.000^**^
Heart	D_mean_ [Gy]	3.9 ± 1.1	3.6 ± 1.0	0.476
Heart	V_17 Gy_ [%]	1.3 ± 1.4	0.8 ± 1.1	0.261
LAD	D_mean_ [Gy]	7.0 ± 2.2	5.7 ± 1.5	0.034^**^
LAD	D_2%_ [Gy]	14.9 ± 3.7	12.3 ± 3.8	0.037^**^
Spinal cord	D_max_ [Gy]	16.9 ± 3.3	18.7 ± 3.8	0.215
Ipsilateral lung	D_mean_ [Gy]	11.2 ± 1.1	10.5 ± 1.1	0.069
Ipsilateral lung	V_17 Gy_ [%]	25.8 ± 3.6	23.0 ± 3.1	0.017^**^
Contralateral lung	D_mean_ [Gy]	3.4 ± 0.4	4.2 ± 0.8	0.003^**^
Contralateral breast	D_mean_ [Gy]	3.0 ± 0.8	4.4 ± 0.9	0.000^**^

Among the 20 patients analyzed (median follow-up, 2 months), no grade ≥3 acute side effect was observed. The most frequent adverse event was radiation-induced dermatitis, with 12 patients (60%) experiencing grade 1 and 2 patients (10%) experiencing grade 2; the remaining 6 patients (30%) had no skin side effect. Acute esophagitis was less frequent, limited to grade 1 in 3 patients (15%), while all others remained asymptomatic.

## 4 Discussion

This study demonstrated the feasibility and safety of automated DIBH breast radiotherapy on a ring-gantry platform using an integrated surface-guided gating system. To our knowledge, this is the first clinical report of an automated DIBH workflow on a ring-based accelerator. Treatment delivery was technically robust, with consistent target coverage, reliable normal tissue sparing, and acute adverse events limited to grade 1–2 events at a median follow-up of two months. Importantly, feasibility was confirmed in patients requiring complex locoregional irradiation, including internal mammary and supraclavicular nodes, showing that the approach is not restricted to simple whole-breast cases and is equally applicable to simpler scenarios.

DIBH techniques have evolved from manual beam-holds to automated solutions, with SGRT now considered the most robust method [15], [16], [20]. Most reports concern C-arm LINACs, where automation is available but gantry geometry can increase collision risk [23], [24]. Ring-based LINACs mitigate this but have until now only supported manual DIBH [25], [26], [27], [28]. The device studied in this manuscript integrated surface guidance and automated gating that provides an intrinsic safety benefit. In manual DIBH workflows, beam-hold decisions rely on the therapist’s continuous visual monitoring, which introduces the possibility of human-factor errors such as delayed reaction time, missed threshold excursions, or premature beam re-activation; events repeatedly documented in incident-reporting systems (*e.g.*, ROSIS) and emphasized in safety reports [42], [43], [44], [45], [46]. By contrast, the studied tool applies predefined geometric thresholds with automatic beam on/off control, eliminating subjective interpretation and ensuring that irradiation only occurs when the patient is strictly within the gating window [32]. This automation reduces cognitive load for radiation therapists, enhances consistency across fractions, and may contribute to a safer and more reproducible DIBH delivery, particularly in treatments requiring multiple breath-holds per session [29].

In the evaluated system, in-bore surface monitoring was performed using a single centrally located camera. Although this may miss certain regions, prior work showed that camera occlusion and couch motion minimally affect DIBH accuracy [30]. The technical performance and reproducibility of the automated gating system, including sub-millimetric motion accuracy and latency measurements, were previously validated in our dedicated end-to-end study [32]. The present clinical study complemented these findings by showing that the system could be effectively combined with a fixed-beam IMRT delivery mode. In our cohort, treatment plans most frequently employed six beams in tangential orientations, with additional oblique fields selectively added to improve nodal coverage. Mean PTV V_95%_ consistently remained above 97% for low-risk volumes and above 99% for boost volumes, while HI was within clinically acceptable ranges. At the same time, OOI objectives were met in all patients: the mean heart dose remained below 4 Gy, LAD doses averaged 7 Gy with maximum values <20 Gy, ipsilateral lung V_17 Gy_ was limited to 25%, and contralateral lung and breast exposure remained very low.

Workflow analysis further confirmed that the approach is clinically manageable. Acquisition of the daily kVCT positioning scan required 20–32 s, depending on the craniocaudal length of the treated volume, and was consistently feasible in DIBH. The median planned beam-on time was 230 s, while the median delivered beam-on time was 416 s, corresponding to a ratio of 1.8 across all fractions. Fig. 3 illustrates patient-specific variability, with notably patient #8 showing higher values due to individual difficulties in sustaining longer breath-holds during certain sessions; patient #18 also underwent bilateral irradiation, further contributing to longer delivery times. Despite this increase, overall treatment efficiency was preserved, with a median fraction duration of approximately 10 min, including setup, imaging, and delivery. This duration was comparable to DIBH workflows reported on conventional C-arm linacs, and remained compatible with standard clinical, although inter-patient variability in breath-hold performance should be anticipated when scheduling treatment slots [19], [47], [48], [49]. The median duration of individual beam-on segments was 18.5 s (range, 0.8–69.3 s), a duration well tolerated by patients and consistent with the reproducibility observed during training. Actual breath-holds were often longer, but this metric specifically reflects the effective intervals during which irradiation was delivered, as there is no need to maintain deep inspiration during gantry rotation or beam preparation. Finally, the large number of recorded gating events (>3500) confirms that the system performed reliably and that patients were able to comply with repeated breath-hold cycles throughout their treatment courses. In addition, the surface guidance system continuously monitored the full 3D thoracic surface during each breath-hold, allowing automatic detection of non-reproducible patterns such as arching, hunching, or shifts from thoracic to abdominal breathing. This real-time surface tracking ensured that irradiation was delivered only when the patient remained within the predefined DIBH geometry, thereby supporting breath-hold consistency throughout the course.

A theoretical comparison with helical delivery in DIBH mode indicated a set of trade-offs: helical mode slightly reduced cardiac doses and improved low-risk PTV HI, while fixed-beam IMRT significantly lowered contralateral exposure. Currently, the fully automated workflow is only available with IMRT; extending it to helical delivery would combine rotational dosimetric benefits with automated gating. Beyond this perspective, fixed-beam IMRT may offer practical advantages such as enabling skin flash directly in the optimizer, avoiding the need for a virtual bolus, while helical delivery remains inherently robust with respect to respiratory motion and breast inflammation. Although a dosimetric comparison between DIBH and free-breathing plans was performed for both fixed-beam IMRT and helical delivery as part of our institutional quality-assurance process, this analysis was not included here as it lies outside the primary scope of this feasibility-focused study. Together, these elements, combined with the reduced collision risk and operational simplicity of ring-gantry systems, support a smooth transition for centers moving from C-arm–based DIBH workflows to fully automated gating on ring-gantry platforms. A full comparison of heart–lung trade-offs, including integral dose considerations, lies beyond the scope of this feasibility-focused study.

Acute side effects in our series were limited to grade 1–2 events, with no patient experiencing grade ≥3 reactions. Radiation-induced dermatitis was observed in 10% of patients at grade 2, while the remainder had grade 0–1, and acute esophagitis occurred in 15% of patients, restricted to grade 1. These rates lie at the lower end of those reported in the literature for moderately hypofractionated breast radiotherapy including regional nodal irradiation, where grade ≥2 dermatitis is typically observed in 10–30% of patients [50], [51], [52], [53]. They are also consistent with the recent findings of the HypoG-01 phase III trial, in which 40 Gy in 15 fractions was associated with 14.3% grade ≥2 dermatitis and very low rates of esophageal adverse events (<3%) [54]. The absence of grade ≥2 esophagitis and the low incidence of clinically relevant skin reactions in our cohort suggest that combining hypofractionation with fully automated DIBH-guided IMRT can achieve a favorable side effect profile, in a cohort of patients requiring complex locoregional target volumes.

In summary, this study provided the first clinical evidence that fully automated DIBH breast cancer radiotherapy can be safely delivered on a ring-based accelerator. Fixed-beam IMRT plans achieved robust target coverage and organ-at-risk sparing, while workflow metrics confirmed that the approach is compatible with routine practice, with clinically acceptable treatment sessions durations and patient compliance supported by short, reproducible breath-holds. Acute side effects were limited to grade 1–2 events, consistent with or below the rates reported in large hypofractionation trials. Importantly, feasibility was demonstrated in locoregional treatments including internal mammary and supraclavicular nodes, indicating that the technique is equally applicable to simpler whole-breast irradiations. Together, these findings highlight automated DIBH with IMRT on a ring-gantry system as a safe and clinically valuable solution for modern breast cancer radiotherapy, with potential for further extension to rotational delivery in the future.

## CRediT authorship contribution statement

**Sarra Midani:** Investigation, Data curation, Formal analysis, Writing – original draft, Writing – review & editing. **Paul Retif:** Investigation, Conceptualization, Methodology, Resources, Data curation, Formal analysis, Writing – original draft, Writing – review & editing, Visualization, Supervision, Project administration. **Sébastien Maksimovic:** Data curation, Writing – review & editing. **Clémence Bondue:** Investigation, Writing – review & editing. **Mohammed Yacoubi:** Investigation, Writing – review & editing. **Gianandrea Pietta:** Investigation, Writing – review & editing. **Anwar Al Salah:** Investigation, Writing – review & editing. **Estelle Pfletschinger:** Investigation, Writing – review & editing. **Motchy Saleh:** Investigation, Writing – review & editing. **Abdourahamane Djibo Sidikou:** Investigation, Writing – review & editing. **Romain Letellier:** Investigation, Writing – review & editing. **Fabian Taesch:** Investigation, Writing – review & editing. **Emilie Verrecchia-Ramos:** Writing – review & editing. **Xavier Michel:** Investigation, Conceptualization, Methodology, Resources, Validation, Supervision, Project administration, Writing – review & editing.

## Declaration of competing interest

The authors declare that they have no known competing financial interests or personal relationships that could have appeared to influence the work reported in this paper.
