# Supplementary material for: Feasibility and workflow efficiency of automated deep inspiration breath-hold for locoregional breast irradiation on a ring-gantry accelerator
Source: Phys Imaging Radiat Oncol. 2026 Jan 13;37:100904. doi: 10.1016/j.phro.2026.100904 (PMC12830249; doi:10.1016/j.phro.2026.100904)
Supplement: Supplementary Data 1 [file mmc1.pdf]

SUPPLEMENTARY FIGURE S1

Representative isodoses of patient #19 in axial, coronal and sagittal planes for the same DIBH setup in helical (left) and fixed-beam IMRT (right) plans.

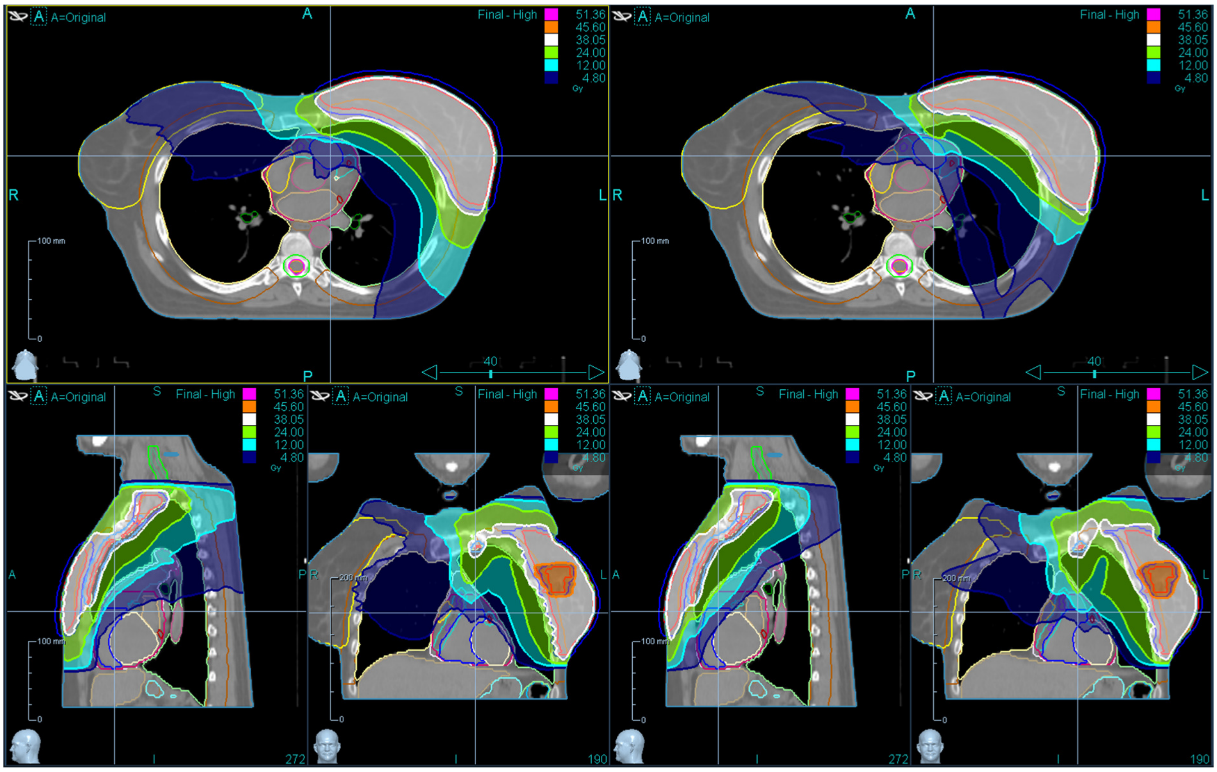

SUPPLEMENTARY TABLE S1

Dose constraints applied to target volumes and organs at risk during treatment planning.

**Structure**	**Metric**	**Objective**
PTV	$V_{95\%}$ [%]	$\geq 95\%$
PTV	$V_{107\%}$ [%]	$\leq 2\%$
PTV	D_{max} [%]	$\leq 110\%$
Heart	D_{mean} [Gy]	≤ 6 Gy
Heart	$V_{17\ Gy}$ [%]	$\leq 10\%$
LAD	D_{mean} [Gy]	≤ 12 Gy
LAD	$D_{2\%}$ [Gy]	≤ 24 Gy
Spinal cord	D_{max} [Gy]	≤ 31 Gy
Ipsilateral lung	D_{mean} [Gy]	≤ 12 Gy
Ipsilateral lung	$V_{17\ Gy}$ [%]	$\leq 25\%$
Contralateral lung	D_{mean} [Gy]	≤ 4 Gy
Contralateral breast	D_{mean} [Gy]	≤ 4 Gy
